# A Petunia Homeodomain-Leucine Zipper Protein, PhHD-Zip, Plays an Important Role in Flower Senescence

**DOI:** 10.1371/journal.pone.0088320

**Published:** 2014-02-14

**Authors:** Xiaoxiao Chang, Linda Donnelly, Daoyang Sun, Jingping Rao, Michael S. Reid, Cai-Zhong Jiang

**Affiliations:** 1 Department of Horticulture, Northwest A&F University, Yangling, Shaanxi, China; 2 Department of Plant Sciences, University of California Davis, Davis, California, United States of America; 3 Crops Pathology and Genetic Research Unit, United States Department of Agriculture, Agricultural Research Service, Davis, California, United States of America; Ecole Normale Superieure, France

## Abstract

Flower senescence is initiated by developmental and environmental signals, and regulated by gene transcription. A homeodomain-leucine zipper transcription factor, *PhHD-Zip*, is up-regulated during petunia flower senescence. Virus-induced gene silencing of *PhHD-Zip* extended flower life by 20% both in unpollinated and pollinated flowers. Silencing *PhHD-Zip* also dramatically reduced ethylene production and the abundance of transcripts of genes involved in ethylene (*ACS*, *ACO*), and ABA (*NCED*) biosynthesis. Abundance of transcripts of senescence-related genes (*SAG12*, *SAG29*) was also dramatically reduced in the silenced flowers. Over-expression of *PhHD-Zip* accelerated petunia flower senescence. Furthermore, *PhHD-Zip* transcript abundance in petunia flowers was increased by application of hormones (ethylene, ABA) and abiotic stresses (dehydration, NaCl and cold). Our results suggest that PhHD-Zip plays an important role in regulating petunia flower senescence.

## Introduction

The onset of flower senescence is known to be initiated by plant hormones, including ethylene, cytokinins and abscisic acid (ABA) [1]–[4]. Flower senescence in petunia and other ethylene-sensitive flowers, is associated with a climacteric rise in ethylene production [5]–[7] and application of ethylene accelerates corolla senescence and the expression of senescence-related genes. Senescence can significantly be delayed by treating flowers with ethylene action inhibitors, such as silver thiosulfate (STS) and 1-methylcyclopropene (1-MCP) [5], [8]–[9]. However, even in ethylene sensitive flowers, other plant hormones also play important roles. A senescence-associated fall in cytokinins and concomitant rise in ABA levels has been demonstrated in several species, including petunia [10]–[13]. The transcriptional basis for the regulation of the synthesis and response to different hormones during flower senescence is still poorly understood.

Detailed analysis of *Arabidopsis* petal senescence data [14] revealed that the transcription factor (TF) families specifically up-regulated in petals were AP2-EREBP, homeobox (HB) and AUX-IAA. Among the HB TFs up-regulated in petals was KNAT1, a member of the Class I KNOX family known to modulate cytokinin levels [15]. A MADS box TF: FOREVER YOUNG FLOWER (FYF/AGL42) acts as a repressor of ethylene-mediated floral abscission and senescence in *Arabidopsis* [16]. Ethylene-responsive element binding factor (ERF) family genes were studied in detail in petunia flowers [17]. Group VII ERFs, known to be involved in fruit ripening were found to be associated with petunia corolla senescence [18]–[19]. In four-o’clock (*Mirabilis jalapa),* flower senescence was associated with strong up-regulation of a homeodomain-leucine zipper TF (HD-Zip) [20].

The HD-Zip family transcription factors are unique to plants and contain a leucine zipper motif (LZ) immediately downstream of the homeodomain (HD). HD-Zip proteins are classified into four subfamilies, I–IV, based on the conservation of the HD-Zip domain, gene structure, additional conserved motifs and functions [21]. Subfamily I (HD-Zip I) proteins are ∼35 kDa in size, and have a highly conserved HD DNA-binding domain without additional conserved motifs besides the Zip domain [21]. Proteins encoded by *HD-Zip I* genes form dimers that recognize *in vitro* the pseudopalindromic sequence CAATNATTG [22]–[25]. HD-Zip I transcription factors play an important role in the regulation of development in response to changes in environmental conditions and hormonal stimuli, especially under water deficit stress and different light conditions [26]. However the function of HD-Zip I genes in flower senescence hasn’t previously been investigated.

Petunia is an ideal model system for studies of flower senescence because of its short life cycle, large corolla, and amenity to biochemical and molecular analysis [27]. We previously used virus-induced gene silencing (VIGS) using a tobacco rattle virus (TRV) vector to investigate the function of senescence-related genes in petunia corollas [28]. A cluster of genes highly expressed during development and senescence of petunia flower were identified [29], including several transcription factors that were up-regulated in senescent corollas [29]. One of them belongs to HD-Zip family, named PhHD-Zip. We report here that silencing this gene, using VIGS in petunia extends flower life, and that over-expressing it accelerates flower senescence. The results suggest a role for PhHD-Zip in regulating flower senescence.

## Results

### A Petunia *HD-Zip* Homolog, *PhHD-Zip* is Highly Expressed during Flower Senescence

**Figure 1:**
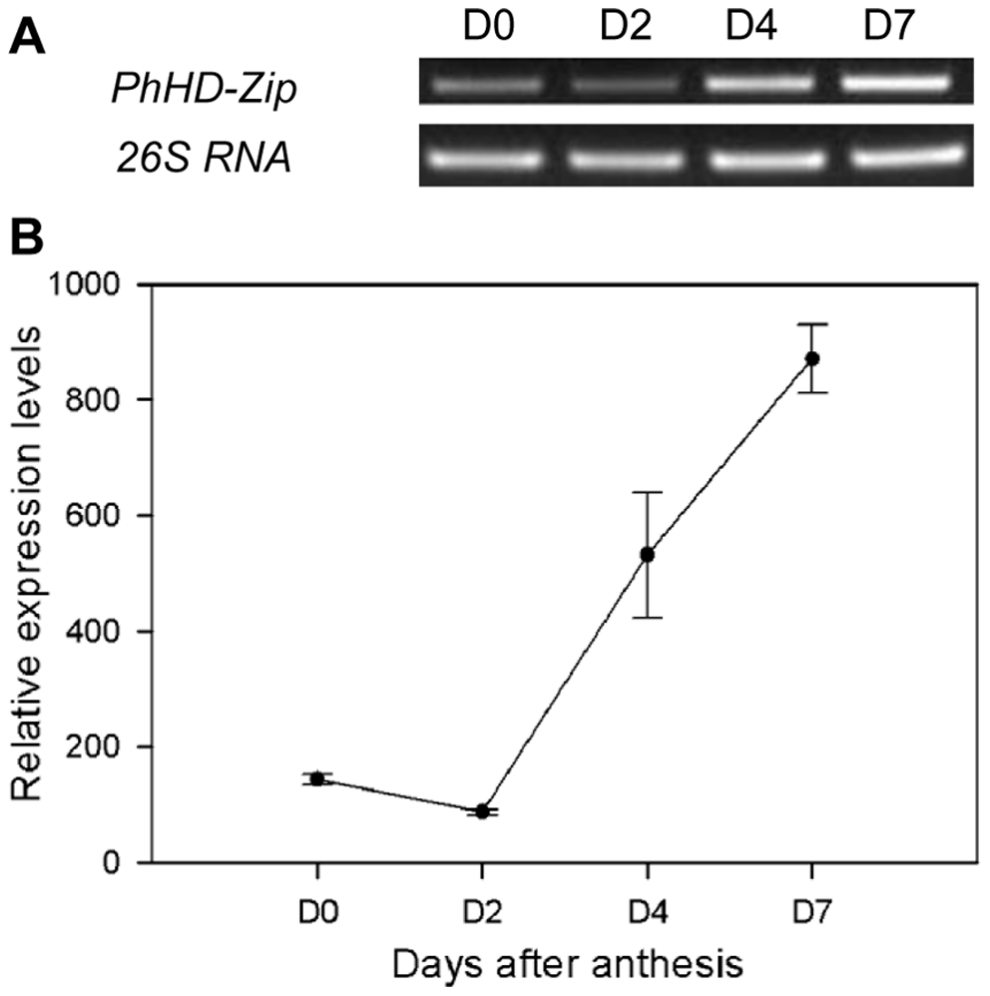
Expression of *PhHD-Zip* in petunia corollas during flower senescence. **A.** A representative gel image from semi-quantitative PCR of RNA isolated from corollas harvested at intervals after anthesis. D0: at anthesis; D2, D4, D7: 2, 4, and 7 days after anthesis, respectively. 26S RNA: the internal control. Samples were analyzed after 30 cycles of amplification for *PhHD-Zip*, and after 24 cycles of amplification for *26S RNA*. **B.** Relative expression levels of *PhHD-Zip* (quantification of the gel pictures; error bars show SE of the means of three biological replicates).

**Figure 2:**
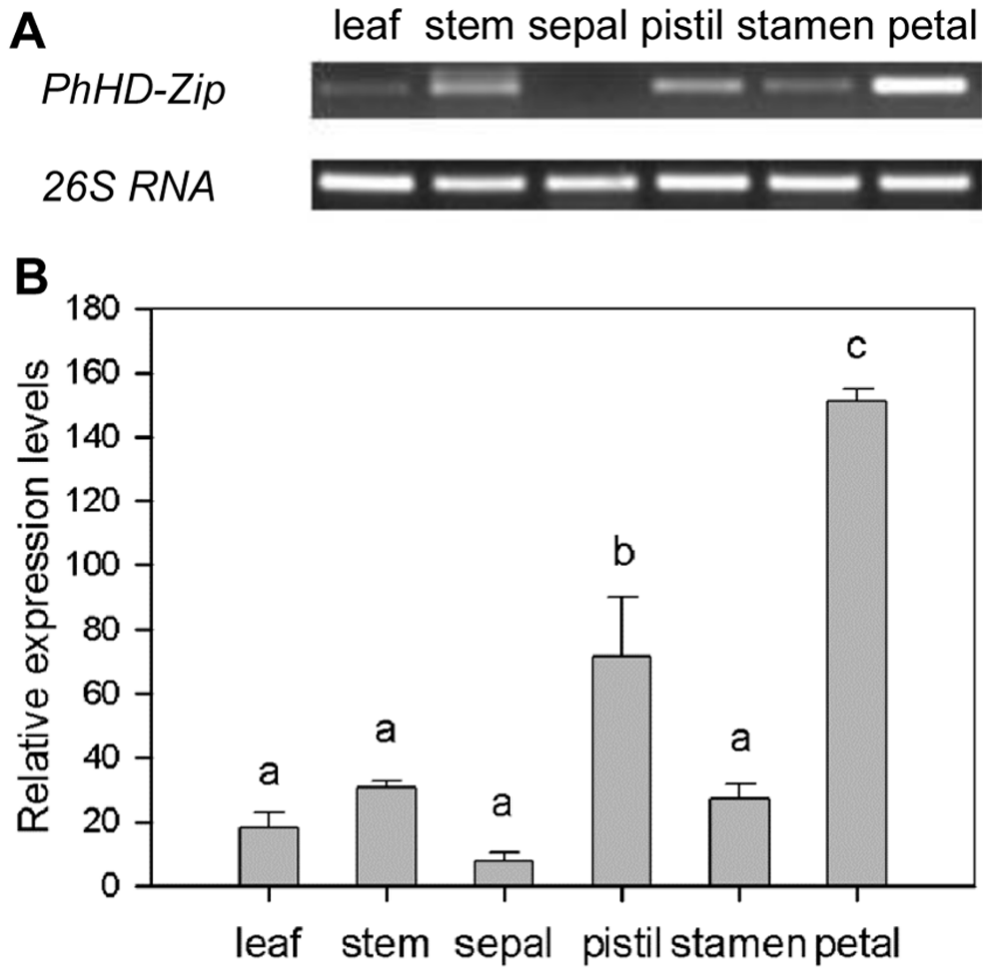
Expression of *PhHD-Zip* in different tissues of petunia. **A.** A representative gel image from semi-quantitative PCR of RNA isolated from different tissues. 26S RNA: the internal control. Samples were analyzed after 33 cycles for *PhHD-Zip*, and after 24 cycles for *26S RNA*. **B.** Relative expression levels of *PhHD-Zip* in different tissues (quantification of the gel pictures; error bars show SE of the means of three biological replicates; different letters denote significant differences using Duncan’s test at P<0.05).

The *PhHD-Zip* gene (accession number: Ph_TC2830 in the DFCI database) was identified among genes up-regulated during flower senescence, as analyzed using a custom Nimblegen microarray [29]. The array data showed that *PhHD-Zip* transcript abundance increased during flower senescence, with the highest level on day 7 (Fig. S1). Expression analysis using semi-quantitative RT-PCR (Fig. 1) with gene-specific primers confirmed the microarray data. *PhHD-Zip* transcripts were detected in leaf, stem, and all flower organs apart from the sepals, with highest abundance in the corollas (Fig. 2). Analysis of the predicted amino acid sequence of PhHD-Zip by basic local alignment (BLAST) against non-redundant GenBank databases showed that it was highly homologous to the HD-Zips of pepper (CaHD-Zip), tobacco (NaHD20), *Arabidopsis* (ATHB7 and ATHB12) and four o’clock (MjHB-Zip), with identities of 71%, 45%, 41%, 59% and 43%, respectively. Amino acid sequence alignment of these HD-Zip proteins showed that the highest degree of homology was in the HD and Zip domains (Fig. S2). Phylogenetic tree analysis revealed a close relationship of PhHD-Zip to the *Arabidopsis* class I HD-Zips, ATHB7 and ATHB12 (Fig. S3).

### *PhHD-Zip* Expression is Regulated by Ethylene, ABA and other Abiotic Signals

**Figure 3:**
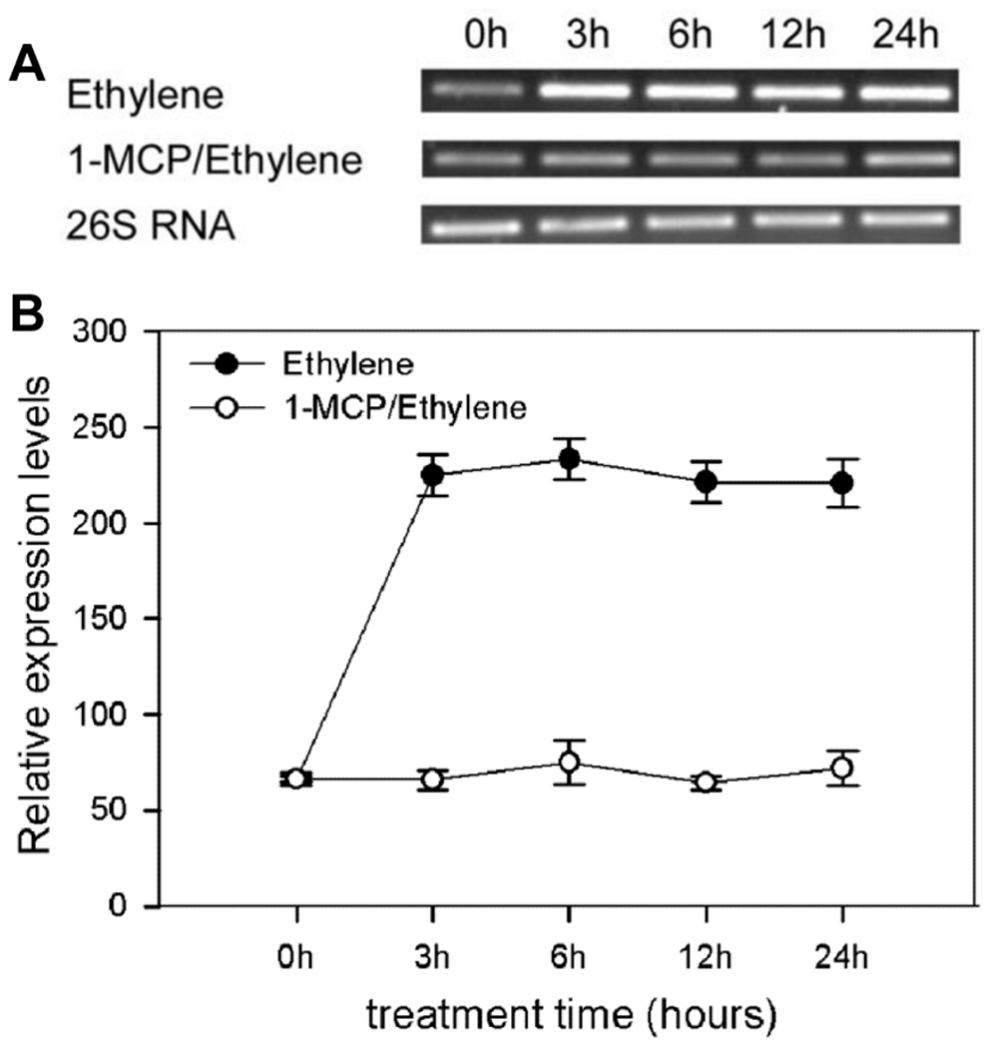
Expression of *PhHD-Zip* in petunia flowers in response to ethylene and 1-MCP treatments. “**Ethylene**” Flowers harvested at anthesis and treated continuously with ethylene (3 ppm), “**1-MCP/Ethylene”** Flowers harvested at anthesis and treated with 1-MCP (50 nL/L) for 4 hours before a continuous ethylene treatment. **A.** A representative gel image from semi-quantitative PCR of RNA isolated from corollas harvested at intervals. 26S RNA: the internal control. Samples were analyzed after 30 cycles for *PhHD-Zip* and after 24 cycles for *26S RNA*. **B.** Relative expression levels of *PhHD-Zip* (quantification of the gel pictures; error bars show SE of the means of three biological replicates).

Ethylene plays a major role in regulating petunia flower senescence. To examine whether *PhHD-Zip* responds to ethylene, detached WT flowers were treated with ethylene and 1-MCP, an ethylene action inhibitor. The expression of *PhHD-Zip* was induced by ethylene 3 h after the treatment, and then remained at high abundance throughout the treatment (Fig. 3). Ethylene-induced expression was clearly prevented by pre-treatment with 1-MCP (Fig. 3).

**Figure 4:**
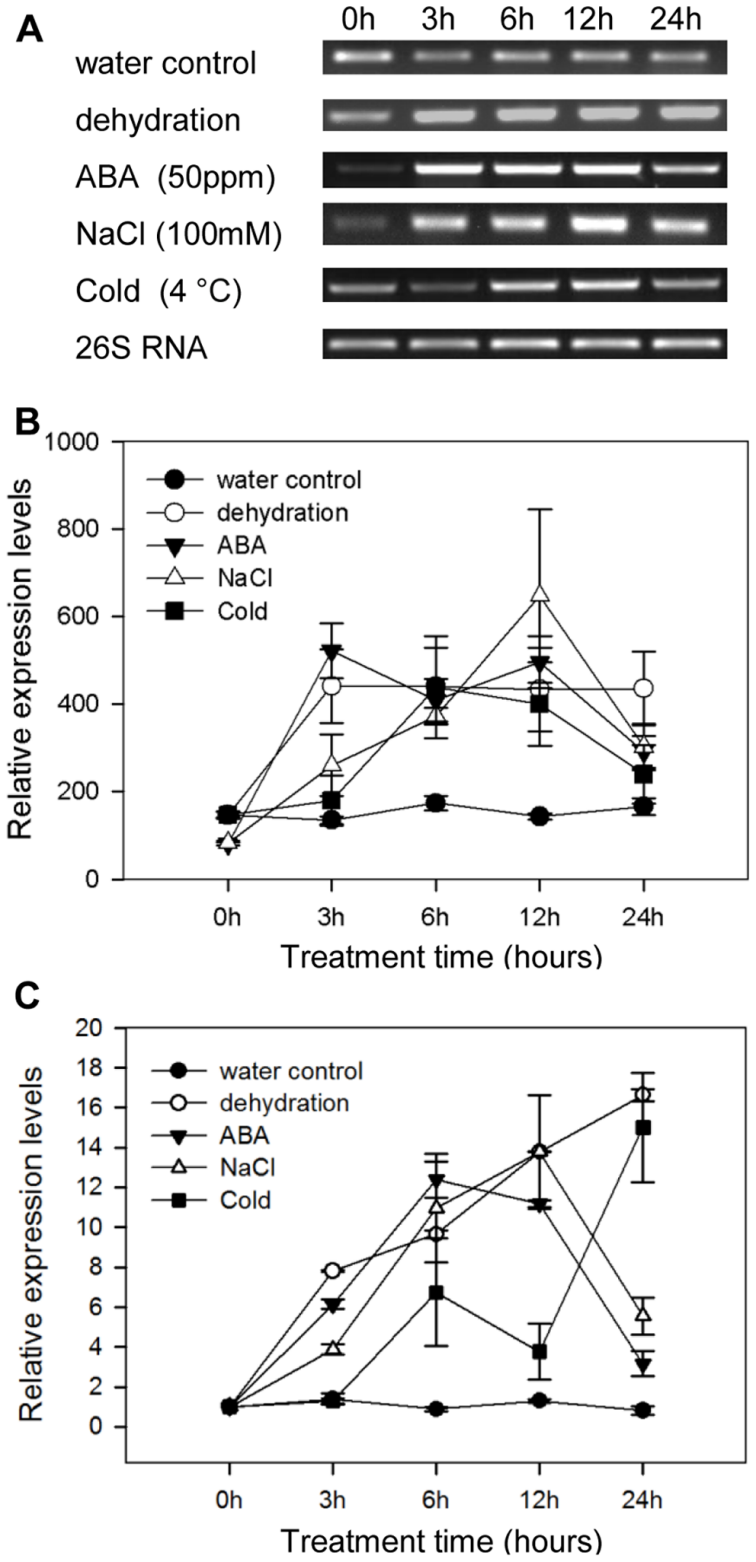
Expression of *PhHD-Zip* in petunia flower under abiotic stress. Petunia flowers harvested at anthesis were placed in tubes with water, without water, with 50°C. **A.** A representative gel image from semi-quantitative PCR of RNA isolated from corollas harvested at intervals. 26S RNA: the internal control. Samples were analyzed after 30 cycles for *PhHD-Zip,* and after 24 cycles for *26S RNA.* **B.** Relative expression levels of *PhHD-Zip* (quantification of the gel pictures; error bars show SE of the means of three biological replicates). **C.** Relative expression levels of *PhHD-Zip* determined using the same RNA samples, but using real-time quantitative PCR (error bars correspond to SE of the means of three biological replicates).

The homologs of *PhHD-Zip* in other species are known to be induced by abiotic stresses like dehydration, salt, and cold [30]–[32]. Detached flowers were placed under stress conditions or treated with ABA or NaCl. Semi-quantitative RT-PCR was conducted to analyze transcript abundance of *PhHD-Zip*. Expression of *PhHD-Zip* was induced by dehydration, ABA, NaCl and cold treatment (Fig. 4A, B). These results were confirmed with real-time quantitative RT-PCR (Fig. 4C).

### Silencing *PhHD-Zip* Extends Flower Longevity

**Figure 5:**
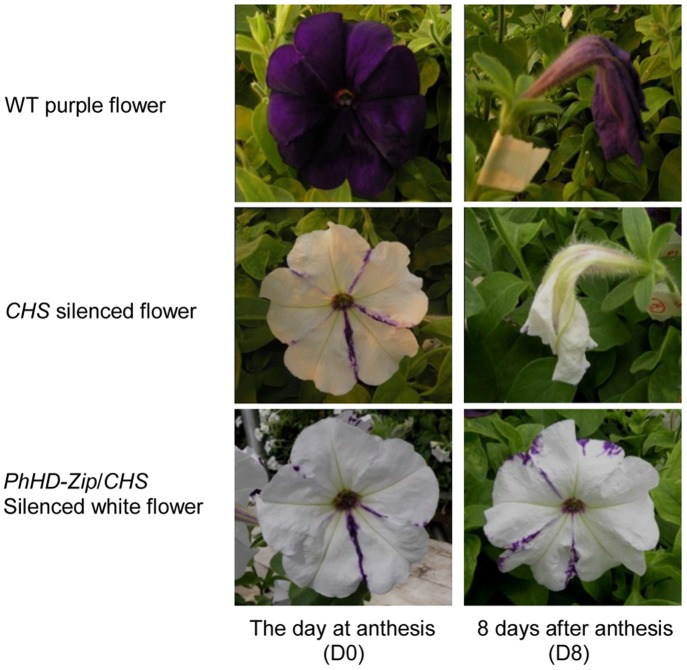
Effect of silencing *PhHD-Zip* on longevity of petunia flowers. Photographs were taken on D0 and D8 of attached purple control flowers (WT), white (silenced) flowers of plants inoculated with the *CHS*/TRV reporter construct, and white (silenced) flowers of plants inoculated with the *PhHD-Zip*/*CHS*/TRV silencing construct.

To examine the role of *PhHD-Zip* in flower senescence, TRV-based VIGS technology was used to down-regulate the expression of this gene. A 287 bp fragment of *PhHD-Zip* was cloned into a silencing construct bearing a fragment of petunia chalcone synthase (CHS) gene as a visual reporter [28]. The transcript abundance of *PhHD-Zip* was greatly reduced in silenced flowers (*PhHD-Zip*/*CHS*/TRV white flower) compared with WT and vector controls (VW, PhHD-Zip (P)) (Fig. S4). Silencing *PhHD-Zip* increased flower longevity by more than one day (approximately 20%) in comparison to the controls - WT flowers, flowers from the vector control and unsilenced purple flowers from plants inoculated with the *PhHD-Zip*/*CHS*/TRV silencing construct (Table 1 and Fig. 5). Silencing *PhHD-Zip* also significantly extended the life of pollinated flowers (Table 1).

**Table 1:** Longevity of unpollinated and pollinated WT, *PhHD-Zip* silenced white flowers, and vector control flowers (*CHS* silenced white, PhHD-Zip/CHS purple).

	Natural Longevity (days±SD)	Pollinated Longevity (days±SD)
WT purple flowers	8.4^a^±1.1	3.4^a^±0.4
*CHS* white flowers	8.2^a^±1.3	3.2^a^±0.5
*PhHD-Zip/CHS* purple flowers	8.2^a^±1.5	3.4^a^±0.2
*PhHD-Zip/CHS* white flowers	9.7^b^±1.7	4.3^b^±1.0

Longevity of attached flowers. Means±SD for 30 flowers (10 from each of three replicate plants). Different letters indicate significant differences using Duncan’s test, p<0.05.

Under drought conditions, the longevity of *PhHD-Zip* silenced flowers (*PhHD-ZIP/CHS* white flower) was one day more than WT and vector control (*CHS* white flowers) for both detached flowers and flowers on the plant (Table 2).

**Table 2:** Effect of drought on longevity of WT, *CHS* silenced control and the *PhHD-Zip* silenced petunia flowers.

	Detached flowers (days±SD)	Attached flowers (days±SD)
WT purple flowers	6.4^a^±0.8	7.1^a^±0.6
*CHS* white flowers	6.2^a^±1.1	7.5^a^±0.5
*PhHD-Zip/CHS* white flowers	7.4^b^±1.0	8.6^b^±1.1

Means±SD for 15 detached flowers (5 from each of 3 replicate plants) and 30 attached flowers (10 from each of 3 replicate plants. Different letters indicate significant differences using Duncan’s test, p<0.05.

### Silencing *PhHD-Zip* Reduces Ethylene Production and the Abundance of Senescence-related Genes

**Figure 6:**
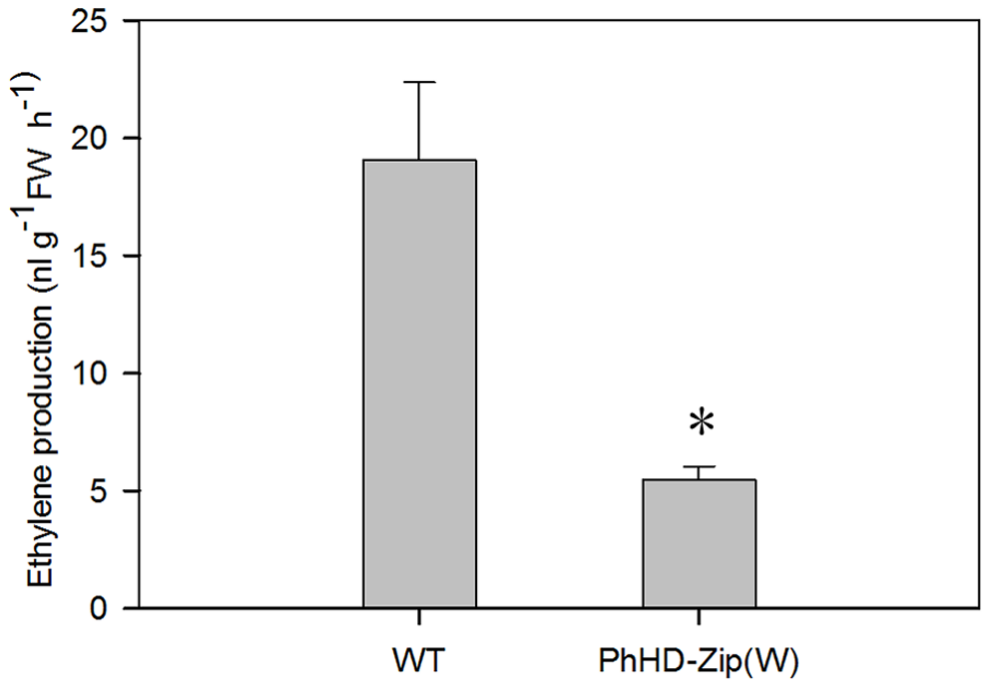
Effect of silencing *PhHD-Zip* on ethylene production of petunia flowers. Ethylene production was measured at D7 for wild type (WT) and for silenced (white) flowers (PhHD-Zip W). (Asterisks denote statistical difference using Duncan’s test at P<0.05; n = 5, error bars denote SE of the means of five biological replicates).

**Figure 7:**
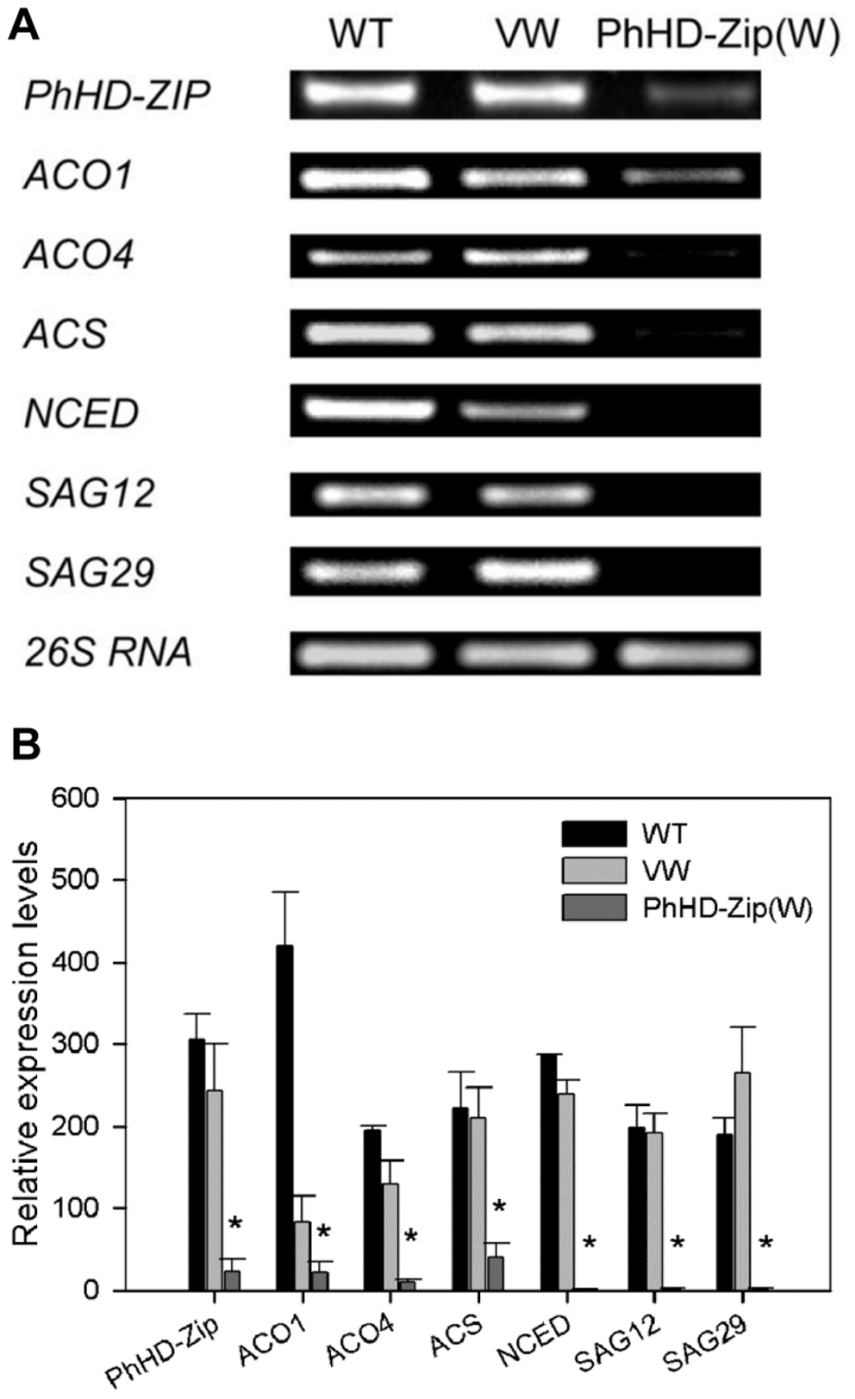
Expression of senescence-related genes in D7 petunia flowers. Abundance of transcripts of genes associated with senescence were determined at D7 in purple control flowers (WT), in white flowers of plants inoculated with the *CHS*/TRV reporter construct (VW), and in white flowers of plant inoculated with the *PhHD-Zip*/*CHS*/TRV silencing construct. **A.** A representative gel image from semi-quantitative PCR of RNA isolated from corollas. 26S RNA: the internal control. Samples were analyzed after 33 cycles for ACS, after 30 cycles for other genes, and after 24 cycles for *26S RNA*, respectively. **B.** Relative expression levels of different genes (quantification of the gel pictures; error bars show SE of the means of three biological replicates; different letters denote significant differences using Duncan’s test at P<0.05).

Ethylene production on day 7 was significantly reduced in the *PhHD-Zip* silenced flowers (Fig. 6), as was the abundance of transcripts of the ethylene biosynthesis genes *ACO1*, *ACO4* and *ACS* (Fig. 7). Transcript abundance of *NCED* (9-cis-epoxycarotenoid dioxygenase), a key enzyme in the ABA biosynthesis pathway, was also reduced in *PhHD-Zip* silenced flowers (Fig. 7). Petunia homologs of the senescence-associated genes, *SAG12* and *SAG29,* were highly expressed in WT and vector control flowers, but were undetectable in *PhHD-Zip* silenced D7 flowers (Fig. 7).

### Over-expression of *PhHD-Zip* Accelerates Petunia Flower Senescence

**Figure 8:**
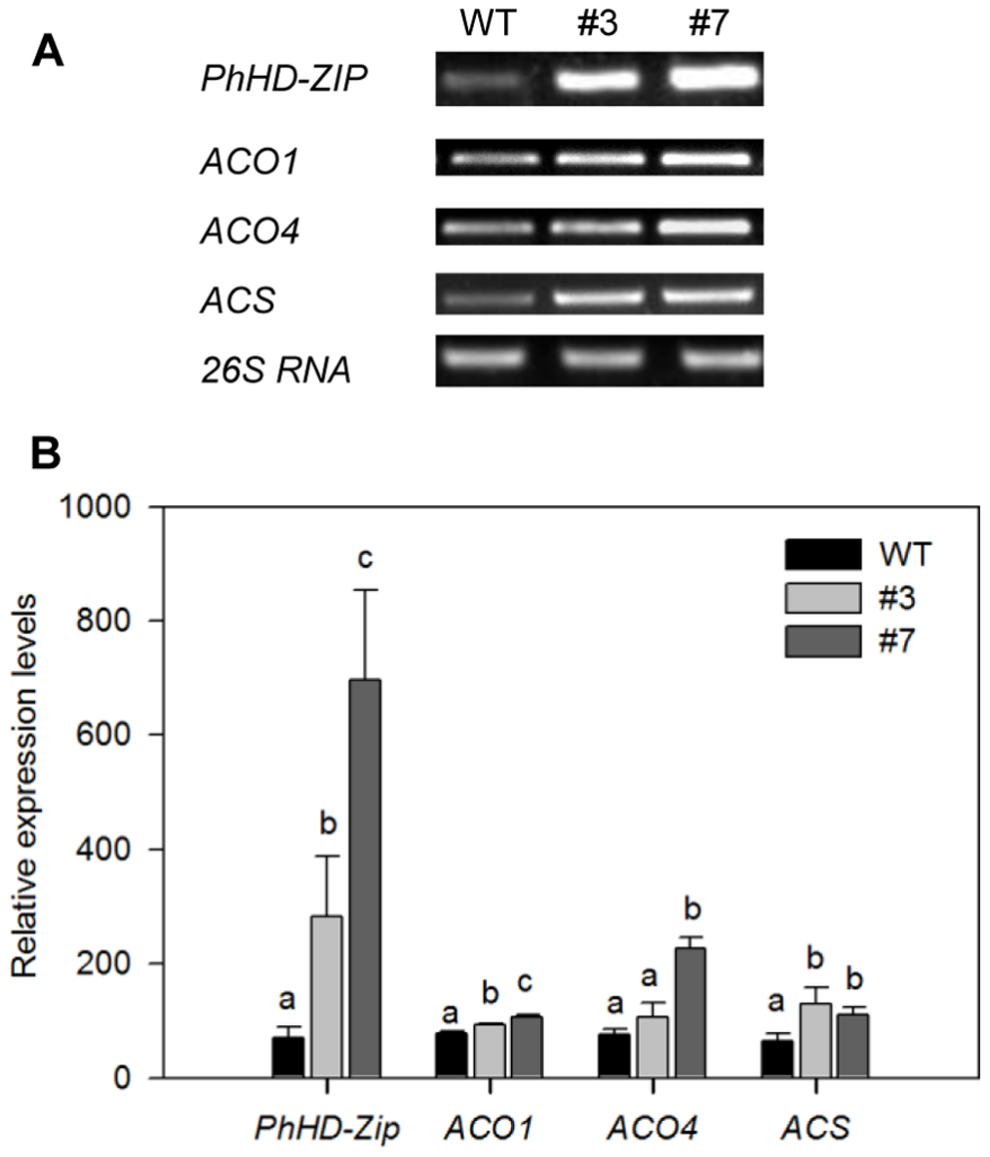
Effect of transgenic over-expression of *PhHD-Zip* on abundance of transcripts of *PhHD-Zip* and of ethylene biosynthesis genes in petunia corollas. WT: wild type flower; #3, #7: two transgenic lines of 35S::*PhHD-Zip*. **A.** A representative gel image from semi-quantitative PCR of RNA isolated from harvested corollas. 26S RNA: the internal control. Samples were analyzed after 30 cycles for *PhHD-Zip, ACO1* and *ACO4*; after 33 cycles for *ACS*; and after 24 cycles for *26S RNA*. **B.** Relative expression level of *PhHD-Zip* and ethylene biosynthesis genes (quantification of the gel pictures; error bars show SE of the means of three biological replicates; different letters denote significant differences using Duncan’s test at P<0.05).

We transformed petunia plants with a full-length sequence of *PhHD-Zip* driven by the constitutive CaMV 35S promoter. Transgenic plants had smaller leaves and flowers (Fig. S5) and a strongly increased expression of *PhHD-Zip* even at anthesis (D0). The two lines (#3 and #7) selected for further analysis showed accelerated flower senescence (Table 3) and increased abundance of transcripts of genes (*ACO1*, *ACO4* and *ACS)* associated with ethylene biosynthesis (Fig. 8).

**Table 3:** Longevity of attached flowers from WT plants and from plants over-expressing *PhHD-Zip* (35S::*PhHD-Zip* lines: #3 and #7).

	Longevity (days±SD)
Wild-type	6.9^a^±0.6
35S::*PhHD-Zip* #3	6.2^b^±0.4
35S::*PhHD-Zip* #7	6.0^b^±0.4

Different letters denote significant differences using Duncan’s test, p<0.05.

## Discussion

### PhHD-Zip is a Transcription Factor Associated with Flower Senescence

In petunia, ethylene plays a key role in flower senescence; exposure to ethylene accelerates senescence, and treatment with inhibitors of ethylene biosynthesis or action extends flower life [33]. However, as in other flowers, the timing of senescence in petunia is modulated by a range of hormonal and environmental signals. The effect of these diverse signals is likely regulated by transcription factors that modify the synthesis and/or response to ethylene. Studies in *Arabidopsis* have indicated the participation of transcription factors from several different families in petal senescence [14]. Interestingly, the *Arabidopsis* data do not suggest a role for members of the HD-Zip family. In previous studies with the model plant four-o’clock, we found a dramatic increase in abundance of transcripts of an HD-Zip homolog [20]. In order to test the hypothesis that HD-Zip TFs may be involved in floral senescence, we elected to work with the more easily manipulated petunia model system.

The high expression of an *HD-Zip* homolog in our petunia microarray analysis mirrored the results that we had obtained from four-o’clock, so we isolated and characterized the gene, which we named *PhHD-Zip*. Using multiple alignment and phylogenetic tree analysis, we identified the gene as a class I *HD-Zip* TF, closely related to *Arabidopsis ATHB7* and *ATHB12*. These TFs are usually associated with responses to abiotic stress, including the ABA response system [30]–[31], [34]. Increased abundance of *ATHB5* transcripts in transgenic *Arabidopsis* plants causes an enhanced sensitivity to the inhibitory effect of ABA on seed germination and seedling growth [35]. T-DNA insertion lines of *ATHB12* in *Arabidopsis* show reduced sensitivity to ABA, whereas over-expression lines of *ATHB12* show hypersensitivity to ABA in root elongation assays [36].

The expression of HD-Zip I subfamily genes from other species, such as *HaHB4* from sunflower and *NaHD20* from tobacco, is strongly induced by water deficit and ABA [32], [37]. Furthermore, over-expression of sunflower *HaHB4* in *Arabidopsis* delays leaf senescence and reduces ethylene sensitivity, suggesting that its encoding protein could serve as a new component of ethylene signaling pathways [38]. Silencing a tomato HD-Zip gene, *LeHB-1*, reduces *LeACO1* mRNA level in tomato fruits and inhibits ripening [39].

In our experiments, the abundance of *PhHD-Zip* transcripts mirrored the effects of hormonal and environmental treatments on flower senescence. We tested the hypothesis that *PhHD-Zip* might play a regulatory role in flower senescence by silencing and overexpression experiments. The results are consistent with an important, but not gate keeping role in the progression of senescence. Down-regulation of *PhHD-Zip* expression by VIGS resulted in a 20% extension of flower life (Table 1), while over-expression under the control of the constitutive 35S promoter shortened it by 15% (Table 3). A search of the public petunia gene sequence database surfaced a 485 bp sequence (Ph_TC9847) with 81% identity to *Arabidopsis ATHB7* and only 65% identity to *PhHD-Zip*. Whether Ph_TC9847 has a function in floral senescence, perhaps having functional redundancy with *PhHD-Zip* remains to be elucidated. The presence of a redundant TF would be consistent with the relatively modest extension of flower life achieved by silencing *PhHD-Zip*. However, if HD-Zip TFs are central regulators of ethylene-mediated flower senescence, we might have expected a more dramatic acceleration of senescence in the lines over-expressing *PhHD-Zip*.

**Figure 9:**
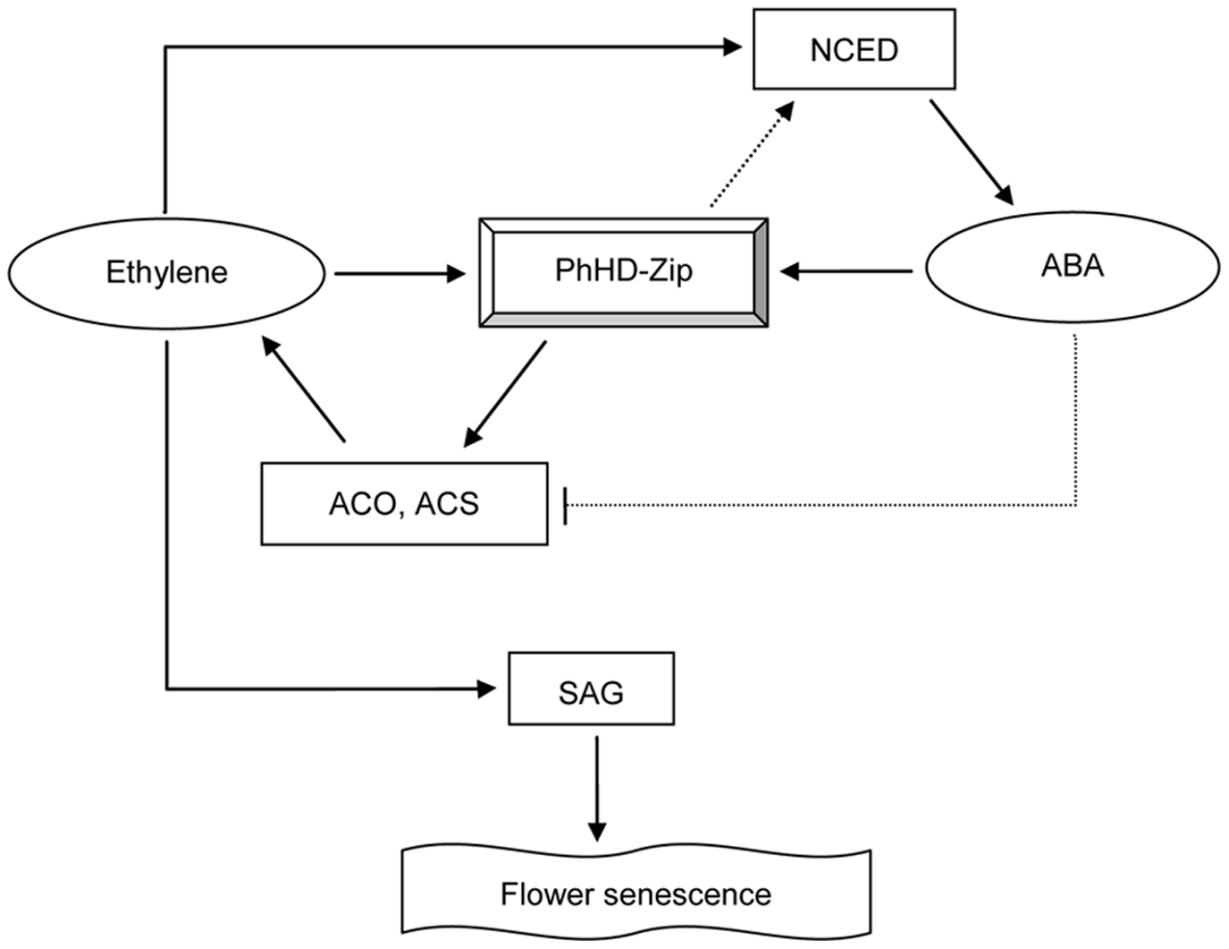
The model of PhHD-Zip regulating flower senescence. Solid lines denote relationships supported by our study and those of other researchers; dashed lines denote hypothetical relationships.

The question of how *PhHD-Zip* might be involved in regulation of flower life was addressed by investigating its interaction with the ethylene pathway. Application of ethylene, which dramatically shortens flower life, increased *PhHD-Zip* abundance. These responses were both prevented by pre-treatment with the ethylene binding-site inhibitor 1-MCP. Clearly, therefore, up-regulation of *PhHD-Zip* by ethylene requires the action of the ethylene response cascade. On the other hand, silencing or overexpression of *PhHD-Zip* reduced or increased, respectively, ethylene production and the abundance of transcripts of genes required for ethylene biosynthesis (Figs. 6–8). These data suggest a feedback loop, where increases in ethylene production increases *PhHD-Zip* abundance and vice versa. Such a model (Fig. 9) is supported by the effects of pollination, where the normal rapid senescence, attributed to an upsurge in ethylene production [40] was delayed in flowers where *PhHD-Zip* was silenced (Table 1). In untreated flowers, *PhHD-Zip* transcript abundance increases before the onset of the ethylene climacteric, so it appears reasonable to hypothesize that PhHD-Zip may be involved in initiating ethylene biosynthesis and consequent senescence events during natural senescence (Fig. 1) [41]–[42]. A search for potential HD-Zip binding motifs revealed the presence of the HD-Zip like motif CAATTATTA and TAATNATTA, and the consensus binding site TAATTA for homeodomain proteins [43] in the 1.5 Kb-upstream promoter regions of the petunia *ACS1*, *ACS2* and *ACO1* genes (Table S1).

### *PhHD-Zip* May Mediate Crosstalk between Ethylene and ABA during Flower Senescence

ABA is thought to play an important role in regulating senescence of some flowers, and may be a key regulator in ethylene-insensitive flowers such as daylily [44], although its role in daffodil and four-o’clock is less certain [45]. In ethylene-sensitive flowers, ABA can accelerate senescence. In rose and hibiscus, ethylene and ABA both stimulate senescence, but ABA treatment suppresses ethylene production [46]. In contrast, the accelerated senescence of carnations caused by ABA is due to stimulation of ethylene synthesis [47].

In petunia, ethylene is recognized as the primary positive regulator of flower senescence, and ABA is a down-stream product; blocking ethylene signaling in transgenic petunia expressing *etr1-1* abolished ABA accumulation in senescing corollas [48]. In support of this conclusion, we have recently demonstrated that over-expression of *NCED* (the key enzyme in ABA biosynthesis) has little effect on flower senescence (A. Estrada, M.S. Reid and C.-Z. Jiang, unpublished). In *Arabidopsis,* HD-Zip TFs are postulated to play an important role in stress responses, in which ABA is also known to play an important regulatory role. In the present study we showed that *PhHD-Zip* transcript abundance was up-regulated by stress and senescence effectors, and it seems possible that PhHD-Zip may play a role in the cross-talk between ethylene and ABA during petunia flower senescence. Expression level of *PhHD-Zip* was up-regulated both by ethylene (Fig. 3) and by ABA (Fig. 4). Silencing *PhHD-Zip* by VIGS down-regulated abundance of transcripts of ethylene biosynthesis genes and the ABA biosynthesis gene (*NCED*) (Fig. 7), and reduced ethylene production (Fig. 6), while over-expression of *PhHD-Zip* up-regulated their levels (Fig. 8). Furthermore, *NCED* transcript abundance was up-regulated by ethylene and down-regulated by 1-MCP (Fig. S6). As diagrammed in Fig. 9, *PhHD-Zip* may be an intermediate in ethylene and ABA crosstalk, positively regulating ABA production. Although ABA does not accelerate petunia flower senescence it may, as suggested for daffodil [45] play a coordinating role in the later stages of senescence. ABA may also play a role in modulating ethylene biosynthesis. The antagonistic interaction between ethylene and ABA has been well-documented in *Arabidopsis* and other species during seed germination and seedling growth [49]–[50]. ABA reduced accumulation of ACO transcripts and ethylene production [49], [51]. On the other hand, ethylene negatively regulates ABA biosynthesis and signaling [52]–[53]. Likewise, higher abundances of ACO and ACS transcripts were demonstrated in ABA-deficient mutant [49]–[50], [54]. Consistent with this finding, ABA-deficient mutants of *Arabidopsis* (*aba2*) and of tomato (*flacca* and *notabilis)* have higher rates of ethylene production [55]–[56]. Our model (Fig. 9) draws on these results to suggest that ABA may play a role in regulating ethylene production. However, whether this antagonistic interaction between ABA and ethylene occurs during petunia flower senescence requires elucidation (Fig. 9).

## Materials and Methods

### Plant Materials and Growth Conditions

Petunia (*Petunia × hybrida*, ‘Primetime Blue’ and *Petunia × hybrida, ‘*Mitchell Diploid’) plants were used in this study. Petunia seeds were obtained from Goldsmith Seeds (Gilroy, CA, USA). Four week old seedlings of *Primetime Blue* were inoculated with the VIGS vector. After inoculation, the seedlings were maintained in the greenhouse or growth chamber with a day (16 h)/night temperature regime of 25/20°C. Seedlings of *Mitchell Diploid* to be used for transformation studies were grown in the greenhouse under the same conditions.

Tissues of petunia *Primetime Blue* used for analyzing *PhHD-Zip* expression were collected from 12-week-old plants. The fully expanded leaves were collected from the top of the plant. The stems were collected between the 2^nd^ and the 3^rd^ leaves from the top of the branch. The pistils and stamens were collected prior to anther dehiscence. The sepals and petals were collected from flowers at anthesis. During flower senescence, flower petals were collected at anthesis (D0), and 2 days (D2), 4 days (D4) and 7 days (D7) after anthesis. D7 petals from wild type, vector control and *PhHD-Zip* silenced flowers were used to determine the abundance of transcripts of *PhHD-Zip* and senescence-related genes.

### Hormones and Abiotic Stress Treatments

The whole flower and pedicel was cut from the plant prior to anther dehiscence but after corolla opening. Three individual flowers were used for each treatment. The flowers were placed immediately in tubes with distilled water, 50 ppm ABA or 100 mM NaCl at room temperature. For cold treatment, flowers were placed in tubes with distilled water at 4°C. For ethylene treatment, flowers were placed in tubes with distilled water, then sealed in a large glass container and treated with 3 ppm ethylene. Corollas were collected before treatment (0 h) and after 3, 6, 12 and 24 h. For 1-MCP treatment, flowers were put in tubes with water, then sealed in a large glass container and treated with 50 nL/L 1-MCP for 4 hours. After the 1-MCP treatment the flowers were treated with 3 ppm ethylene; corollas were collected after the 1-MCP treatment (0 h) and after 3, 6, 12, and 24 h of ethylene treatment. Harvested corollas were frozen in liquid nitrogen immediately, then kept at −80°C pending RNA extraction.

### Flower Longevity

Flower longevity studies used at least ten flowers from each of three separate plants. Longevity was recorded as the time from anthesis until the corolla was completely wilted. For the drought treatment, detached flowers were placed in tubes without water for 6 hours; water was then supplied for determination of flower longevity. For attached flowers, plants with flowers were not watered for 36 hours, by which time the leaves were obviously wilting. The plants were re-watered, and the longevity of the flowers was recorded. Statistical analysis of longevity data was performed by JMP10.0 software package (SAS Institute, Cary, NC).

### Identification of *PhHD-Zip*

Total RNA was extracted from petunia corollas using the Trizol Reagent according to the manufacturer’s instructions (Invitrogen, CA, USA). RNA was treated with RNase-free DNase (Promega, Madison, WI, USA) to remove DNA contamination according to the manufacturer’s instructions. 2 µg total RNA was used to synthesis first-strand cDNA using the SuperScript III kit (Invitrogen, CA, USA). The *PhHD-Zip* gene was amplified and cloned using *PhHD-Zip* specific primers (Table S2) under the following PCR conditions: 94°C, 5 min; 94°C 30 s, 58°C 30 s, 72°C 30 s, 35 cycles; 72°C 7 min. The amplified gene was sequenced by the sequencing service of the College of Biological Sciences at UC Davis. Multiple alignments of the *PhHD-Zip* amino acid sequence and phylogenetic tree analysis were performed using the CLUSTALW software online (http://www.genome.jp/tools/clustalw/). Parameters for the multiple alignments were: slow/accurate, multiple gap open penalty 10.0, gap extension penalty 0.1 for pairwise and 0.2 for multiple alignments, protein weight matrix: GONNET. Parameters for phylogenetic tree analysis were: fast/approximate, multiple gap open penalty 10.0, gap extension penalty 0.05, protein weight matrix: BLOSUM, rooted phylogenetic tree: UPGMA.

### VIGS Plasmid Construction

To generate the *CHS*/TRV2 construct, a 194 bp fragment of the *CHS* gene was PCR-amplified from petunia as described previously [28]. The resulting product was cloned into pTRV2 to form the *CHS*/TRV2 construct. To generate the *PhHD-Zip*/*CHS*/TRV2 construct, a 287 bp fragment of *PhHD-Zip* amplified from petunia corolla cDNA using the *PhHD-Zip* gene specific primers F1R1 (Table S2) was cloned into the *CHS*/TRV2 vector. Cultures of *A. tumefaciens* strain GV3101 were transformed with the VIGS plasmids by electroporation. Recombinant *Agrobacterium* clones were selected on LB plates containing 50 µg/mL kanamycin and the presence of the gene fragments was confirmed by PCR.

### Inoculation

*Agrobacterium* clones transformed with RNA1 (pTRV RNA1 construct), and the *CHS*/TRV2 and *PhHD-Zip*/*CHS*/TRV2 constructs were cultured separately in LB media containing 40 mg/L kanamycin, 20 mg/L gentamicin, 10 mM MES and 20 µM acetosyringone for 36∼48 h at 28°C to an OD600 of 2∼4. The *Agrobacterium* cells were harvested by centrifugation at 4000×g for 20 min and resuspended in inoculation buffer (10 mM MgCl_2_, 10 mM MES, 200 µM acetosyringone) and incubated at room temperature with gentle shaking for a minimum of 3 h to an OD600 of 2∼4. The bacteria containing RNA1 and those with modified RNA2 (*CHS*/TRV2 or *PhHD-Zip*/*CHS*/TRV2) were then mixed together in a 1∶1 ratio immediately before inoculation. The RNA1: *CHS*/TRV2 was used as a vector control, and RNA1: *PhHD-Zip*/*CHS*/TRV2 was used as described previously [28] for silencing *PhHD-Zip*.

### Confirmation of Silencing, Semi-quantitative RT-PCR and Real-time Quantitative PCR

Silencing *PhHD-Zip* was monitored by the simultaneous silencing of *CHS*, as shown by visible white spots, sectors, or whole corollas on the normally blue flowers [28]. To confirm silencing, we used a primer pair (F2R2) specific to a region of the gene that did not include the silencing fragment (Table S2). Transcript abundance was determined using semi-quantitative RT-PCR [57]. In brief, 2 µg total RNA was used to synthesize first-strand cDNA with the SuperScript III kit (Invitrogen, CA, USA). PCR was conducted using gene-specific primers (Table S2) and primers for 26S ribosomal RNA that served as an internal control. All sequences were confirmed by the sequencing service of the College of Biological Sciences at UC Davis. To verify the results of the semi-quantitative determination of transcript abundance, real-time quantitative PCR (ABI7300; Applied Biosystem, Foster City, CA, USA) was performed on one set of RNA samples (Fig. 4) using the SYBR Green reagent [57]. Data were analyzed using the 2^−ΔΔCT^ method [58] and are presented as relative levels of gene expression.

### Quantification of Transcript Abundance Semi-quantitative PCR Analyses

The gel pictures from semi-quantitative PCR analyses were quantified using the ImageJ software. The area value of three biological replicates was used to determine relative expression levels. Statistical analysis was performed by one-way ANOVA and SPSS 16.0 for Windows [29]. Duncan’s test was used to examine significance of differences between treatments using a *P* value of <0.05.

### Ethylene Production Measurement

Individual flowers were harvested and incubated at 25°C for 3 h, with their pedicels in a tube of water, in a 250 ml container, filled with sand to reduce the head space (which was measured after the experiment by displacement with water). A 2 ml sample of head-space gas was withdrawn using a gas-tight hypodermic syringe, and injected into a gas chromatograph (GC-8A; Shimadzu, Kyoto, Japan) for ethylene concentration measurement. Measurements are the results from five replicates.

### Over-expression of *PhHD-Zip* and Plant Transformation

A 938 bp DNA sequence containing the ORF region of the *PhHD-Zip* gene was amplified using the *PhHD-Zip-*specific primer pair F3R3 (Table S2). The full-length DNA fragment was cloned into the pGSA1403 vector in the sense orientation, downstream of the CaMV 35S promoter. *A. tumefaciens* strain GV3101 was transformed with the resulting plasmid by electroporation. Transformation and regeneration of petunia *Mitchell Diploid* was performed using the leaf disc method and cultivation process described previously [29]. Flowers used to examine *PhHD-Zip* expression and record longevity were collected from two 35S::*PhHD-Zip* transgenic lines (#3 and #7) in the T0 generation.

## Supporting Information

Figure S1 ***PhHD-Zip*** **transcript abundance during petunia flower senescence.** Total RNA extracted from petunia corollas at intervals during senescence was analyzed using a custom-designed microarray (NimbleGen). Data show transcript abundance normalized to a group of housekeeping genes. D0: at anthesis, D2, D4, D7: 2, 4, and 7 days after anthesis, respectively.
(TIF)

Figure S2 **Alignment of the deduced PhHD-Zip amino acid sequence homologs from other plant species.** CaHD-Zip, Chili pepper (AAQ88401.1), NaHD20, *Nicotiana attenuata* (ADI50265.2), ATHB7, *Arabidopsis* (NP_182191.1), ATHB12, *Arabidopsis* (AEE80275), MjHB-Zip, four o’clock (ACL81158.1).
(TIF)

Figure S3 **Phylogenetic** **comparison of PhHD-Zip with** ***Arabidopsis*** **class I HD-Zips.** The accession numbers of the ATHBs are: ATHB1 (AEE73670.1), ATHB3 (AED92122.1), ATHB5 (AED98037.1), ATHB6 (AEC07305.1), ATHB7 (AEC10740.1), ATHB12 (AEE80275.1), ATHB13 (AEE34974.1), ATHB16 (AEE87163.1), ATHB20 (AEE73626.1), ATHB21 (AEC06780.1), ATHB22 (AEC09273.1), ATHB23 (AEE30764.1), ATHB40 (AEE86696.1), ATHB51 (AED90654.1), ATHB52 (AED96435.1), ATHB53 (AED98253.1), ATHB54 (AEE30774.1).
(TIF)

Figure S4 **Silencing efficiency of VIGS determined by semi-quantitative PCR.** Abundance of *PhHD-Zip* were determined at D6 in purple control flowers (WT), in white flowers of plants inoculated with the *CHS*/TRV reporter construct (VW), and in purple flowers (PhHD-Zip(P)) and white flowers (PhHD-Zip(W)) of plants inoculated with the PhHD-Zip/CHS/TRV construct. **A.** A representative gel image from semi-quantitative PCR of RNA isolated from corollas. 26S RNA: the internal control. Samples were analyzed after 30 cycles *PhHD-Zip* and after 24 cycles for *26S RNA*. **B.** Relative expression level of *PhHD-Zip* as determined by quantification of the gel pictures; error bars show SE of the means of three biological replicates; asterisks denote significant differences using Duncan’s test at P<0.05).
(TIF)

Figure S5 **Phenotype of plants over-expressing** ***PhHD-Zip*** **.** Representative fully-expanded young leaves of WT and 35S::*PhHD-Zip* transgenic petunia lines (#2, #3, #6 and #7). Representative flowers were harvested at anthesis.
(TIF)

Figure S6 **Effects of ethylene and 1-MCP on** ***NCED*** **expression.** Representative gel images from semi-quantitative PCR of RNA isolated from corollas harvested at intervals. **Ethylene:** Flowers harvested at anthesis and treated continuously with ethylene (3 ppm), **1-MCP/Ethylene:** Flowers harvested at anthesis and treated with 1-MCP (50 nL/L) for 4 hours before a continuous ethylene treatment. 26S RNA: the internal control. Samples were analyzed after 30 cycles for *NCED* and after 24 cycles for *26S RNA*.
(TIF)

Table S1 **Occurrence of the listed cis-elements in the 1.5 Kb upstream promoter regions of petunia ACS and ACO genes.**
(DOCX)

Table S2 **Primers used for semi-quantitative RT-PCR.**
(DOCX)
