# Supplementary figures and images for: A Petunia Homeodomain-Leucine Zipper Protein, PhHD-Zip, Plays an Important Role in Flower Senescence
Source: PLoS One. 2014 Feb 14;9(2):e88320. doi: 10.1371/journal.pone.0088320 (PMC3925126; doi:10.1371/journal.pone.0088320)

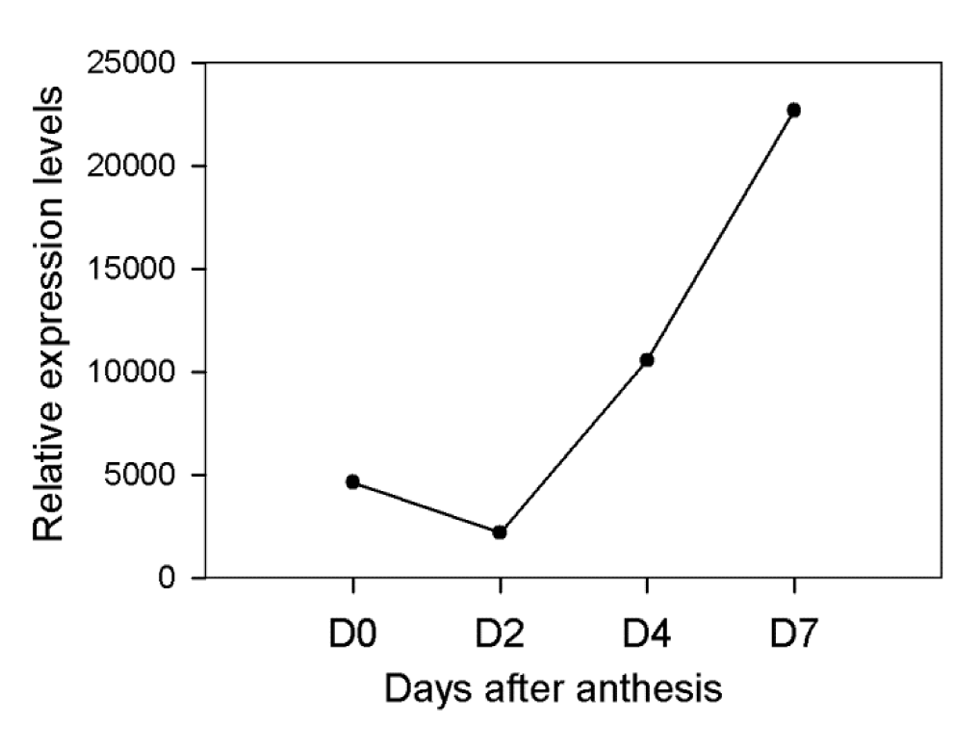

Supplement: Figure S1 — PhHD-Zip transcript abundance during petunia flower senescence. Total RNA extracted from petunia corollas at intervals during senescence was analyzed using a custom-designed microarray (NimbleGen). Data show transcript abundance normalized to a group of housekeeping genes. D0: at anthesis, D2, D4, D7: 2, 4, and 7 days after anthesis, respectively. (TIF) [file pone.0088320.s001.tif]

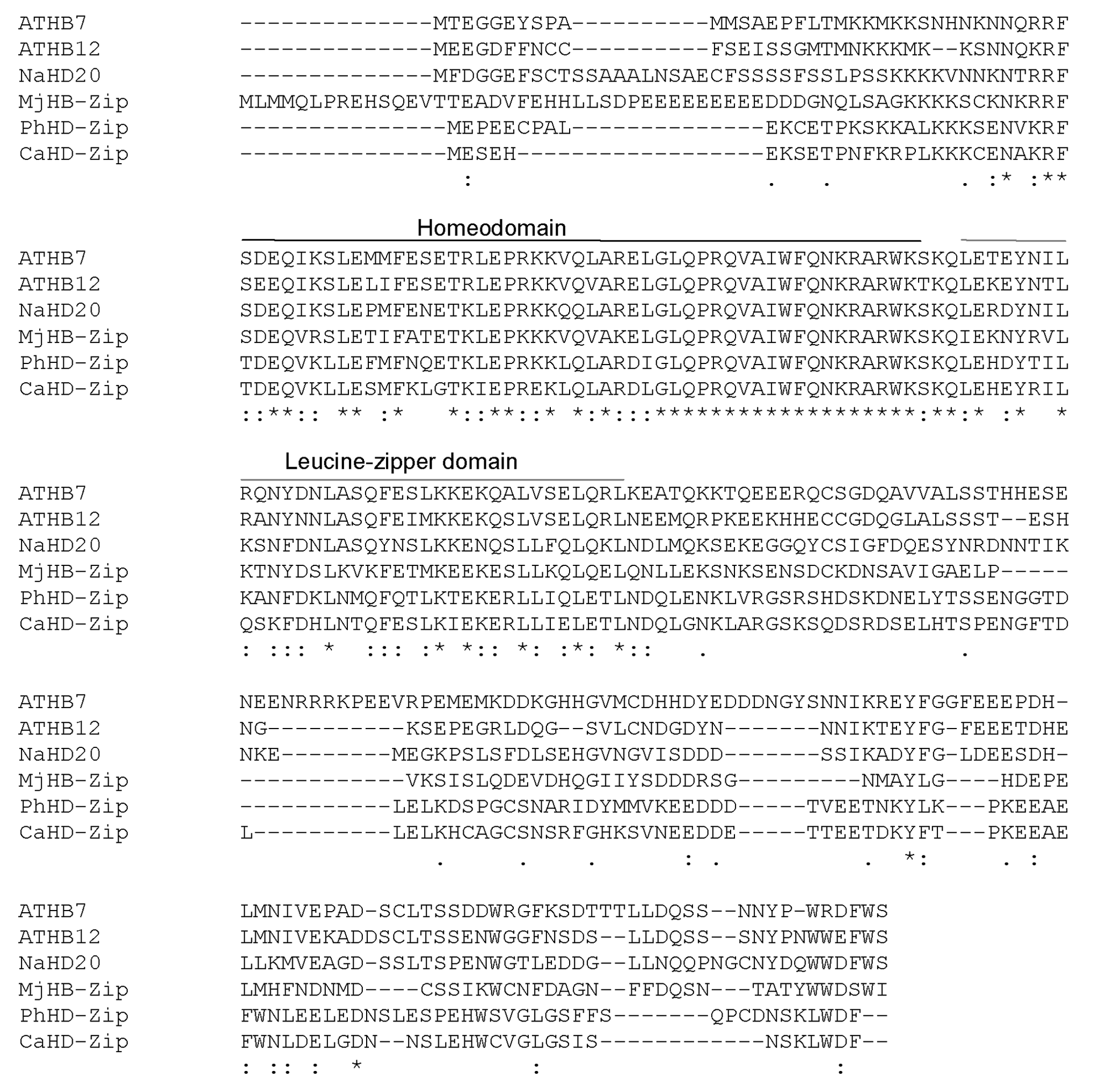

Supplement: Figure S2 — Alignment of the deduced PhHD-Zip amino acid sequence homologs from other plant species. CaHD-Zip, Chili pepper (AAQ88401.1), NaHD20, Nicotiana attenuata (ADI50265.2), ATHB7, Arabidopsis (NP_182191.1), ATHB12, Arabidopsis (AEE80275), MjHB-Zip, four o’clock (ACL81158.1). (TIF) [file pone.0088320.s002.tif]

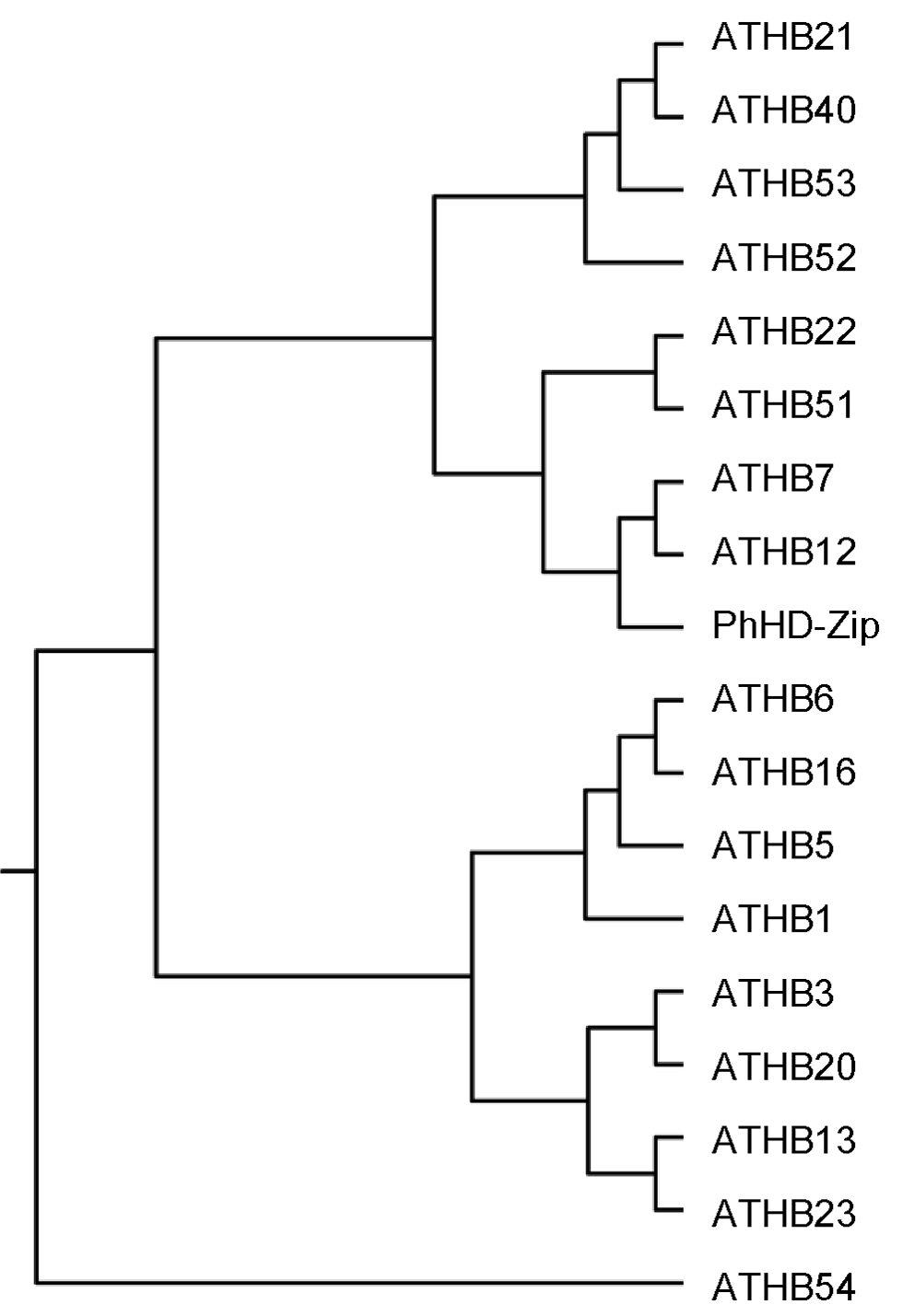

Supplement: Figure S3 — Phylogenetic comparison of PhHD-Zip with Arabidopsis class I HD-Zips. The accession numbers of the ATHBs are: ATHB1 (AEE73670.1), ATHB3 (AED92122.1), ATHB5 (AED98037.1), ATHB6 (AEC07305.1), ATHB7 (AEC10740.1), ATHB12 (AEE80275.1), ATHB13 (AEE34974.1), ATHB16 (AEE87163.1), ATHB20 (AEE73626.1), ATHB21 (AEC06780.1), ATHB22 (AEC09273.1), ATHB23 (AEE30764.1), ATHB40 (AEE86696.1), ATHB51 (AED90654.1), ATHB52 (AED96435.1), ATHB53 (AED98253.1), ATHB54 (AEE30774.1). (TIF) [file pone.0088320.s003.tif]

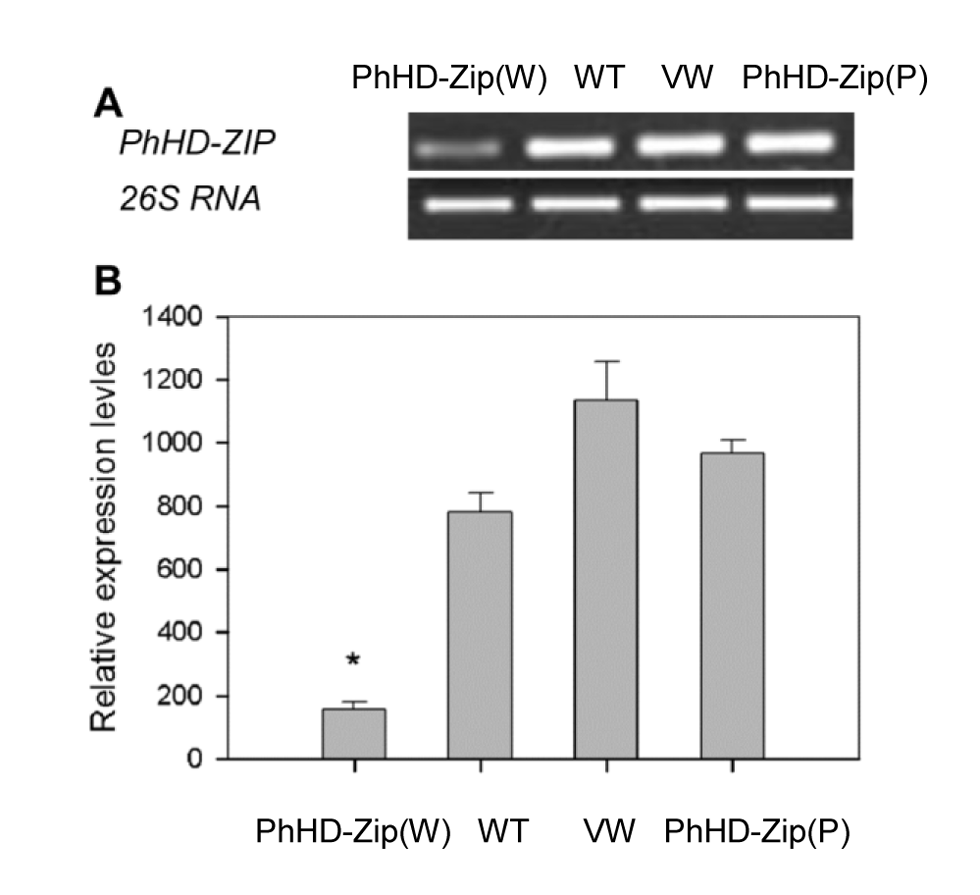

Supplement: Figure S4 — Silencing efficiency of VIGS determined by semi-quantitative PCR. Abundance of PhHD-Zip were determined at D6 in purple control flowers (WT), in white flowers of plants inoculated with the CHS/TRV reporter construct (VW), and in purple flowers (PhHD-Zip(P)) and white flowers (PhHD-Zip(W)) of plants inoculated with the PhHD-Zip/CHS/TRV construct. A. A representative gel image from semi-quantitative PCR of RNA isolated from corollas. 26S RNA: the internal control. Samples were analyzed after 30 cycles PhHD-Zip and after 24 cycles for 26S RNA. B. Relative expression level of PhHD-Zip as determined by quantification of the gel pictures; error bars show SE of the means of three biological replicates; asterisks denote significant differences using Duncan’s test at P<0.05). (TIF) [file pone.0088320.s004.tif]

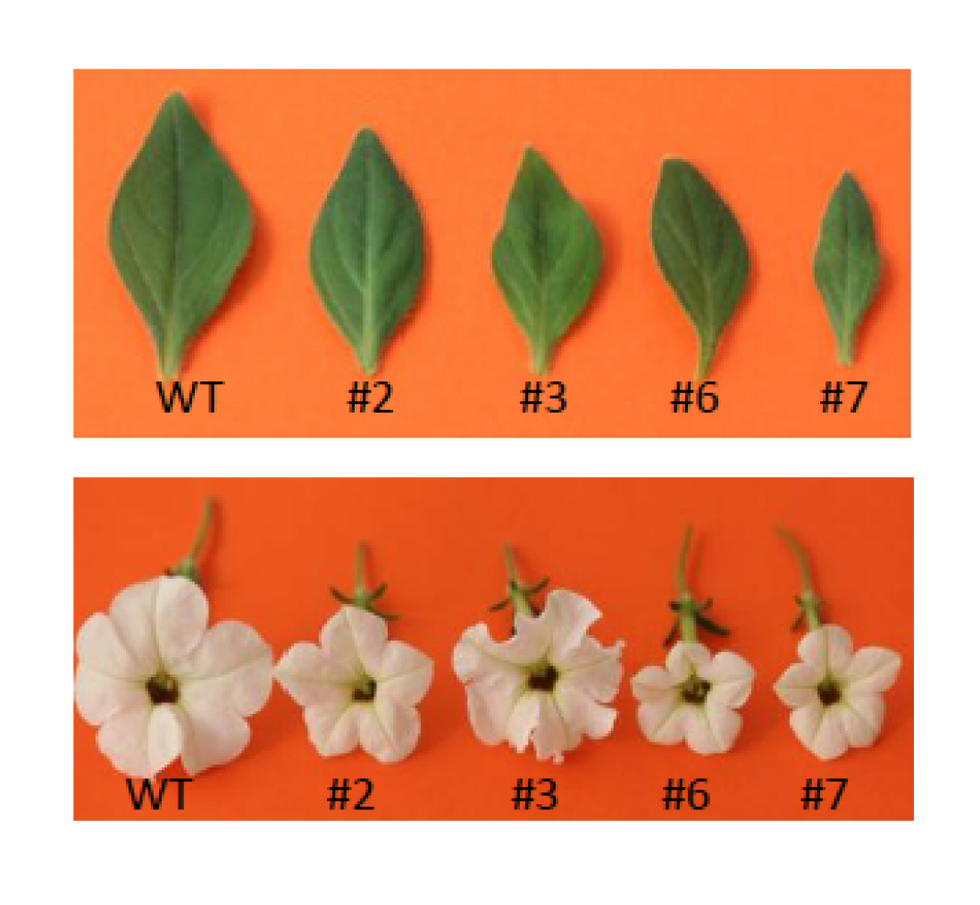

Supplement: Figure S5 — Phenotype of plants over-expressing PhHD-Zip . Representative fully-expanded young leaves of WT and 35S::PhHD-Zip transgenic petunia lines (#2, #3, #6 and #7). Representative flowers were harvested at anthesis. (TIF) [file pone.0088320.s005.tif]

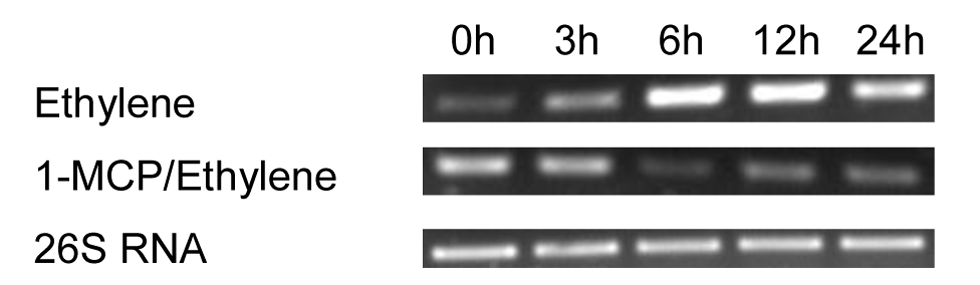

Supplement: Figure S6 — Effects of ethylene and 1-MCP on NCED expression. Representative gel images from semi-quantitative PCR of RNA isolated from corollas harvested at intervals. Ethylene: Flowers harvested at anthesis and treated continuously with ethylene (3 ppm), 1-MCP/Ethylene: Flowers harvested at anthesis and treated with 1-MCP (50 nL/L) for 4 hours before a continuous ethylene treatment. 26S RNA: the internal control. Samples were analyzed after 30 cycles for NCED and after 24 cycles for 26S RNA. (TIF) [file pone.0088320.s006.tif]
