# Supplementary material for: A Petunia Homeodomain-Leucine Zipper Protein, PhHD-Zip, Plays an Important Role in Flower Senescence
Source: PLoS One. 2014 Feb 14;9(2):e88320. doi: 10.1371/journal.pone.0088320 (PMC3925126; doi:10.1371/journal.pone.0088320)
Supplement: Table S1 — Occurrence of the listed cis-elements in the 1.5 Kb upstream promoter regions of petunia ACS and ACO genes. (DOCX) [file pone.0088320.s007.docx]

**Table S1.** **Occurrence of the listed cis-elements in the 1.5 Kb upstream promoter regions of petunia ACS and ACO genes**

| Gene name/NCBI ID | TAATTA | CAATNATTG | CAATTATTA | TAATA/TATTA |
| --- | --- | --- | --- | --- |
| *ACS1*/ U64804.1 | 1 | 0 | 1 | 1 |
| *ACS2*/ AF049711.1 | 1 | 0 | 1 | 0 |
| *ACO1*/ L21976.1 | 1 | 0 | 0 | 2 |
| *ACO4*/ L21979.1 | 0 | 0 | 0 | 0 |
