# Supplementary material for: A Petunia Homeodomain-Leucine Zipper Protein, PhHD-Zip, Plays an Important Role in Flower Senescence
Source: PLoS One. 2014 Feb 14;9(2):e88320. doi: 10.1371/journal.pone.0088320 (PMC3925126; doi:10.1371/journal.pone.0088320)
Supplement: Table S2 — Primers used for semi-quantitative RT-PCR. (DOCX) [file pone.0088320.s008.docx]

**Table S2. Primers used for semi-quantitative RT-PCR**

| Gene ID | Forward primer (5’-3’) | Reverse primer (5’-3’) | Fragment length |
| --- | --- | --- | --- |
| *PhHD-Zip*(F1, R1) | GTTGGAGACATTAAACGAACAGC | CCAGTGTTCAGGTGATTCTAAGG | 287bp |
| *PhHD-Zip*(F2, R2) | AGAGATATTGGCCTGCAACCT | GTCTTTCGAATCGTGGCTTCT | 242bp |
| *PhHD-Zip*(F3, R3) | CCCATATCCAGTCAAATATACAAA | CAAGCTCAGAAATCCCACAACT | 938bp |
| *ACO1* (L21976.1) | GGATCCAATTGCCACTGTCTA | CTGATGGAGAAATGAAGGAACA | 278bp |
| *ACO3* (L21978.1) | AAGCTATGAAGGCAAGGGAAG | CGCAACAAGAGACGGTAACAT | 220bp |
| *ACO4* (L21979.1) | AGAGTGTACCGCACAGAGTGATT | GATCAATCTTCACATCAGCTTCC | 255bp |
| *ACS* (Ph_TC5971) | TTGACTTGATTGAGGACTGGATT | TGCTGGGTAATAAGGTGAAGGTA | 287bp |
| *NCED*(Ph_TC9965) | CGGCAAATCATCTTCAGACAT | ACTGTACATGCCGTCCAATTC | 276bp |
| *SAG12*(Ph_TC1621) | CTCTTGGACACTGCCTTTACATT | AAACACCACTGGAATAGAACTGG | 250bp |
| *SAG29*(Ph_TC9096) | ACTTCTGAGTTGGCTTTCGTCTT | TGGTGGCAAATATCAAGAAGATT | 274bp |
